# Empathy Changes Among Chinese College Students in the Context of Marketization

**DOI:** 10.3390/bs15050597

**Published:** 2025-04-29

**Authors:** Xiaofei Liu, Ziqiang Xin

**Affiliations:** 1 School of Psychology, Inner Mongolia Normal University, Hohhot 010022, China; liuxiaofei1994@foxmail.com; 2 Department of Psychology, School of Educational Sciences, Shanxi Normal University, Taiyuan 030031, China; 3 Department of Psychology, Renmin University of China, Beijing 100872, China

**Keywords:** social change, marketization, empathy, cross-temporal meta-analysis

## Abstract

China’s marketization refers to the systemic reform process of transitioning from a planned economy to a market economy, which has significantly contributed to the country’s economic development. However, the interest-oriented nature of marketization may also somewhat erode social morality. Given that empathy is the basis of morality and prosocial behavior, the present study produced a cross-temporal meta-analysis of 89 studies using the Chinese version of the Interpersonal Reactivity Index as a measuring instrument and outlined the empathy changes among 48,400 Chinese college students from 2009 to 2019. Then, this study tested the conjecture that empathy is weakening in marketization process. The results reveal that Chinese college students’ empathy level declined over time and was negatively correlated with the marketization level. This finding contributes to understanding the relationship between the marketization reform and moral psychology change.

## 1. Introduction

China’s reform and opening-up have led to economic achievements that have attracted worldwide attention. Many Chinese sociological and psychological researchers have experienced these changes and focused on the implications of economic system reform for society (Cai et al., 2020; Huang et al., 2021; J. X. Wang, 2018; Xin, 2019; Zhou, 2017). For instance, Xin (2019) found that while China’s market economy has been rapidly developing, general interpersonal trust has been steadily declining over the years. This phenomenon has been examined through a series of studies, revealing the negative impact of macro-level socio-ecological factors, specifically marketization, on interpersonal trust (Xin, 2019; Z. X. Yang & Xin, 2020). Similarly, Zhou (2017) also observed a general sense of apathy in contemporary Chinese society and argued that this might stem from a lack of sympathy among people due to the changing macro-social ecology.

Zhou’s perspective aligns with the folk theory of social change (Kashima et al., 2009), which suggests that people tend to perceive social transformations as a natural progression—from traditional societies, characterized by communal sharing relationships, to modern societies, dominated by market pricing relationships. Similar social issues, such as moral corruption arising from rapid economic development, were thoroughly discussed in the pioneer countries of the industrial revolution (Horkheimer & Adorno, 1987/2002; Simmel, 1950). Then, the question arises: does marketization, as the most prominent feature of modernization process, indeed correlate with decreased empathy, an antecedent of social morality? To obtain empirical evidence supporting the relationship between the marketization process and decreased empathy, the present study conducted a cross-temporal meta-analysis to explore changes in empathy during modernization process.

### 1.1. Empathy as the Basis of Social Morality

Social morality reflects the widespread natural virtues among individuals in social interaction, such as *ren* [仁] or benevolence. In Confucianism, *Ren* was deemed the core and cornerstone of morality by Confucius and Mencius (Xie, 2019). In the 18th-century British moral philosophy system, benevolence was the original natural virtue emphasized by Hutcheson, Hume, and Smith (Maurer, 2019). Regarding the relationship between empathy and social morality, both Mencius and Smith declared sympathy as the origin of virtues. Sympathy here refers to an instinct in human nature to share and have pity for the misfortunes of others (J. Z. Li, 2021; Smith, 1790/2010).

In psychology, researchers are more accustomed to using the concept of empathy to describe the multifaceted psychological process of imagining someone’s feelings according to their own subjective internal perspective in order to understand others (Davis, 1996; Eklund & Meranius, 2021; Murphy et al., 2022). As a renowned proponent of the multifaceted view of empathy, Davis (1980) developed the Interpersonal Reactivity Index (IRI), which focuses on both the affective (includes personal distress and empathic concern dimensions) and cognitive (includes perspective taking and fantasy dimensions) processes of empathy. This partitioning of empathy consistently continues to this day.

Since then, empathy has become a hot topic in social psychology, and a wealth of evidence has emerged to support empathy as an antecedent of morality (Masto, 2015). Precisely, empathy can positively predict individuals’ altruistic behavior (Batson et al., 2015; Klimecki et al., 2016; Z. X. Li et al., 2019). Moreover, the recent neuroscientific findings on empathy further strengthened the relationship between empathy and prosocial behavior (De Waal & Preston, 2017; Stevens & Taber, 2021; Weisz & Zaki, 2018). In addition to prosocial behavior, some studies have found that empathy can also suppress antisocial behavior and delinquency (Mayer et al., 2018; Trivedi-Bateman & Crook, 2022; Zych et al., 2019). Moreover, empathy is negatively associated with violent crime rates, aggravated assault, and robbery in the United States’ national-level surveys (Bach et al., 2017). In summary, empathy consistently associates with altruistic behavior and negatively predicts deviant behavior. Pieces of evidence from both sides justify empathy as a psychological antecedent of social morality. Referring to the importance of empathy to moral transformation, it is important to reveal how empathy changes over time in a transitional country like China.

### 1.2. Empathy Changes over Time in China

The industrial revolution initiated global modernization, with the transition of production methods bringing about macro-environmental changes in politics, economics, and society, while profoundly affecting individuals’ psychology and behaviors (Greenfield, 2016; Uskul & Oishi, 2020). During China’s modernization, several psychological factors related to social morality have been demonstrated to have significantly changed over time. For instance, interpersonal trust among college students has declined (Z. X. Yang & Xin, 2020), and the moral disengagement levels of middle and university students have consistently decreased (X. K. Wang et al., 2024).

Empathy may also undergo significant changes during the process of social transformation in China. The evidence supports the notion that empathy at the group level is unstable and capable of changing with social shifts. Konrath et al. (2011) conducted a cross-temporal meta-analysis based on the Interpersonal Reactivity Index (IRI) from 1979 to 2009, analyzing a sample of 13,734 U.S. college students. They found a significant decline in both empathic concern and perspective-taking dimensions. Furthermore, a study on empathy among Chinese nurses supports the hypothesis of empathy decline in the context of societal changes in China. Xu et al. (2021) used a cross-temporal meta-analysis (number of samples = 57; total sample size = 13,825) based on the Jefferson Scale of Empathy-Health Professionals to reveal significantly decreased trends in the total empathy and the perspective taking dimension among Chinese nurses during 2009–2018. In sum, these two studies seem to indicate a tendency for empathy to decline over time. However, Yan et al. (2017) conducted a cross-temporal meta-analysis using the Chinese version of the Interpersonal Reactivity Index (number of samples = 18; total sample size = 4082) and found that perspective-taking, personal distress, and total empathy all showed significant upward trends between 2009 and 2015.

In summary, the existing research on changes in empathy in China has yielded contradictory conclusions. Therefore, this study aims to address this inconsistency by using a larger sample size and a broader time span to describe changes in Chinese college students’ empathy. Since cross-temporal meta-analysis is a method for examining variations related to time periods and historical development, it is essential to include as many studies as possible and cover a broad time span. Social-ecological psychology suggests that changes in the macro environment of society may affect individuals’ psychology and behavior (Uskul & Oishi, 2020). Accordingly, the present study also examines the macro-level social factors that co-vary with empathy. The resulting hypothesis is that marketization, as an essential part of modernization, may be related to changes in empathy, as analyzed below.

### 1.3. Marketization as a Macro Societal Change Co-Varies Negatively with Empathy

China’s marketization refers to the systemic reform process transitioning from a planned economy to a market economy (Fan et al., 2011). During the planned economy period, individuals were embedded in collective organizations, such as grassroots neighborhood committees in urban areas and rural people’s communes. Various survival resources were allocated through a planned coupon system. Distribution was managed by grassroots administrative units, including neighborhood committees and communes. This “organizational dependency” mode of production and life is in stark contrast to the independent and autonomous mode under the market economy system. In the market economy, individuals disembed from organizational constraints and develop individual identities. They obtain goods and services necessary for economic activities and daily living through labor markets or commodity markets (Z. X. Yang, 2022). The marketization reform in China can optimize the allocation of resources and factors, improve production and exchange efficiency, promote economic development, and facilitate social progress (M. J. Yang & Luo, 2015). 

However, the effect of marketization on society is not always positive. For example, in American society during the 1990s, severe violent crime was prevalent, which Messner and Rosenfeld (2012), based on the institutional anomie theory (IAT), attributed to the detrimental role of marketization. They claimed that Western capitalist societies’ institutional structures, characterized by economically dominant forms, were imbalanced and further manifested in a culture of anomie value (i.e., a heightened focus on financial success and a disregard for normative constraints), which increased crime rates (Messner et al., 2019). Moreover, the marketized mentality is significantly positively correlated with deviant behaviors such as prejudices, devaluation, and instrumental criminal behavior (Bieliński & Hövermann, 2023; Groß et al., 2018; Hövermann et al., 2015). Bach et al. (2017) examined the associations between state-level empathy, prosocial behavior, and antisocial behavior in the United States. They found that empathy is associated with lower rates of violent crime, aggravated assault, and robbery.

Marketization may also be associated with lower levels of empathy. The following studies further corroborate this conjecture. Some empirical studies showed that market attributes reduce individuals’ concern for other people’s interests and even induce unethical behavior (Falk & Szech, 2013; Reeson & Tisdell, 2010; L. Wang et al., 2014). Although these findings do not directly reveal the relationship between marketization and empathy, such self-interested behavior, namely abandoning morality for profit, is contrary to the altruistic nature of empathy. Furthermore, Molinsky et al. (2012) provided more direct evidence that the subliminal priming of the economic schema reduced individuals’ expressions of compassion for others by partially inhibiting the state of empathy. Based on the above, the present study assumes that empathy may weaken over time within the context of marketization.

### 1.4. The Present Research

Given that China’s marketization has continuously evolved and adapted to the Chinese context over time, it is necessary to assess the cross-cultural applicability of the above assumption. Therefore, this study conducts a cross-temporal meta-analysis of studies that use the IRI-C as an empathy measurement tool to examine trends in empathy changes in China. We then test the hypothesis that empathy has declined during marketization process.

This study focuses on changes in empathy among Chinese college students before 2020, as China’s economy experienced a relatively stable period of development before facing significant disruptions due to the COVID-19 pandemic. College students were selected as the research subjects because studies on empathy among this population are the most abundant, ensuring the availability of at least one paper per year for reference.

## 2. Methods

### 2.1. Database Search and Study Inclusion Rules

The studies on empathy among Chinese college students using the IRI-C as a measurement tool have mainly been published in three forms: Chinese academic theses, Chinese journal articles, and English journal articles. The Chinese journal articles and academic theses can almost all be found in the China National Knowledge Infrastructure (CNKI) and Wanfang Data. The English journal articles were retrieved through Google Scholar. The advantage of using Google Scholar is that it serves as a comprehensive academic search engine, aggregating a wide range of resources (such as those from databases like Scopus, PsycINFO, and Web of Science), including journal articles, theses, books, and conference papers. The search terms included “empathy”, “Interpersonal Reactivity Index”, “IRI-C”, and “college students”, as well as the name of the author who developed the IRI-C (Chan, 1986). The search timeframe was set to include studies published before 31 December 2021. The final database search was conducted on 27 March 2025. The database search obtained 1391 papers, and 7 relevant papers that were found during the reading process were added to reduce missing data, for a total of 1398 papers.

**Figure 1:**
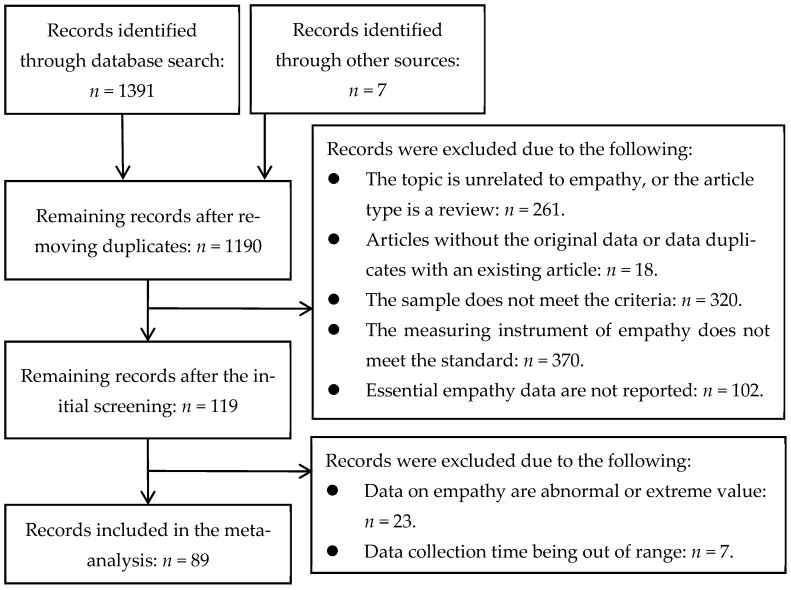
Flow chart of the database search and record screening.

The papers were managed, coded, and screened using the literature management software EndNote X9 by two PhD students studying psychology. The inclusion process is shown in Figure 1.

Initially, the titles and abstracts of the articles were read to exclude those unrelated to the topic of empathy or review articles, while cross-sectional and intervention studies were retained. For the articles that passed this initial screening, the methodology section was carefully examined, and the following criteria were applied for further selection: (1) The sample consisted of college students enrolled in universities in mainland China, with postgraduates excluded, and the students were approximately 18–22 years old. (2) The measurement tool used in each study was the unaltered IRI-C^1^. (3) The IRI-C should use a five-point scale (0–4 or 1–5) ranging from “very inappropriate” to “very appropriate” (Konrath et al., 2011). (4) The essential data on empathy or its dimensions, such as the mean, standard deviation, and sample size, had to be reported. (5) Articles where any dimension score was more than three standard deviations from the mean were excluded, along with those showing anomalous values (e.g., scores higher than 4 on a 0–4 scale or negative values; scores higher than 25 on a 1–5 scale for five items). (6) The article with the earliest publication time and most complete data was selected for different articles that were available within the same database. Ultimately, the present cross-temporal meta-analysis includes 89 studies conducted during 2009–2019, and the chronological distribution of the studies is shown in Table 1.

### 2.2. The Coding of Main Variables

The principles of variable coding are as follows: Background information coding includes the publication class (Chinese core journals = 1, other Chinese journals = 2, doctoral and master dissertations = 3, English journals = 4) and data collection regions (Western region = 0, Middle region = 2, Northeastern region = 3, Eastern region = 4, unreported or cross-regional = 5). In this study, “Chinese core journals” refer to journals listed in the Peking University Chinese Core Journals Database (PKU Core Journals), known for their strong academic influence in China. “Other Chinese journals” are peer-reviewed but have a lower academic impact. If the exact sampling year was not reported in the study, the sampling year was coded as being two years before a journal article’s publication or one year before a dissertation was submitted to a university (Twenge, 2000).

The coding means and standard deviations of empathy and its dimensions included several situations. For studies that used IRI-C with a 1–5 scale, the means of empathy and its dimensions were reduced by 1 to transpose the data to a 0–4 scale. For studies that only reported each dimension’s mean and standard deviation, the mean of total empathy was calculated after being weighted by the number of question items. Moreover, the missing standard deviations were filled by serial means. For studies that only reported the means and standard deviations of sub-studies or sub-groups (e.g., gender, grade level), the mean and standard deviation of the total sample were weighted and synthesized according to the following equations ($\bar{x}$ and $S_{\tau }$ represent the mean and standard deviation of the total sample; $x_{i}$, $s_{i}$, and $n_{i}$ represent the mean, standard deviation, and sample size of the sub-samples).

(1) $$\bar{x}=\sum {x_{i}n}_{i}/\sum n_{i}$$

(2) $$S_{\tau }=\sqrt{\left[\sum n_{i}s_{i}^{2}+\sum n_{i}{\left(x_{i}-\bar{x}\right)}^{2}\right]/\sum n_{i}}$$

### 2.3. Marketization Index

Assessing the level of marketization in a country or region often requires considering multiple aspects of economic development. Researchers have attempted to quantify marketization using various indicators, such as the Economic Freedom of the World Index (Gwartney & Lawson, 2024), which measures the degree of government intervention; the monetization of the economy (X. Li, 2016), which reflects the extent of market-based pricing; and the proportion of private enterprises and self-employed individuals, which indicates the scale of the development of the non-public economy (Lu & Zhu, 2015). To effectively evaluate the differentiated marketization progress across provinces in China, X. L. Wang et al. (2021) developed a comprehensive marketization index for China’s provinces, as published by the Beijing National Economic Research Institute.

The marketization index integrates a wide range of indicators that reflect various aspects of marketization, with a specific focus on five key components: (1) The relationship between the government and the market. This component reflects the dominant role of the market in resource allocation and the extent of government intervention in the economy. A higher score indicates a lower proportion of government-directed resource allocation, less administrative intervention, and a more streamlined government structure. (2) The development of the non-public economy. This component reflects the vitality and scale expansion of the non-state economy in market resource allocation. A higher score indicates a greater share of the non-state economy in income, investment, and employment. (3) The development of the product market. This component reflects the level of marketization in product pricing, competition, and free trade. A higher score indicates a more mature and efficient product market, with greater market-driven price determination and fewer barriers to competition. (4) The development of the factor market. This component reflects the maturity of the market-based allocation for key factors such as finance, human resources, and technological achievements. A higher score indicates more vigorous competition and more efficient resource allocation. (5) The development of market intermediaries and the legal institutional environment. This component reflects the degree of development of market intermediary institutions, the legal environment, and intellectual property protection. A higher score indicates a more sound market operating environment, with better legal protections and service support.

This study utilized the marketization index, following prior research (Xin & Xin, 2017). The data underlying this index came from two sources: provincial-level statistics published by government agencies and data collected through a nationwide enterprise sampling survey. Given the variations in data sources and scope, a standardized methodology was applied to ensure both cross-sectional and longitudinal comparability. The latest *Marketization Index of China’s Provinces: NERI Report* by X. L. Wang et al. (2021) describes the development of marketization in China from 2008 to 2019. The marketization of China experienced a period of stagnation or slow growth (2008–2011), followed by a phase of rapid expansion (2012–2014); after 2015, the growth rate slowed, transitioning into a period of steady development. Despite these fluctuations, the overall trend indicates a continuous increase in marketization levels.

The marketization index was used in this study and obtained from the China Marketization Index Database (https://cmi.ssap.com.cn/dataQuery, accessed on 2 January 2025). To facilitate regional comparisons of the relationship between marketization and empathy from a spatial perspective, this study adopted China’s economic regional classification—the Eastern, Middle, Western, and Northeastern regions. The province-level marketization index was aggregated to the regional level to match the corresponding empathy data collected in each region. Additionally, the national average marketization index was used to correspond to empathy data that were either unreported at the provincial level or collected across multiple regions.

## 3. Results

### 3.1. The Changing Trends in Empathy over Time

This cross-temporal meta-analysis involved a total of 48,400 college students. In order to understand the central tendency and dispersion of empathy between 2009 and 2019 among Chinese college students, the present study performed a descriptive statistics analysis of the means of empathy and each dimension in all the articles (see Table 2).

**Figure 2:**
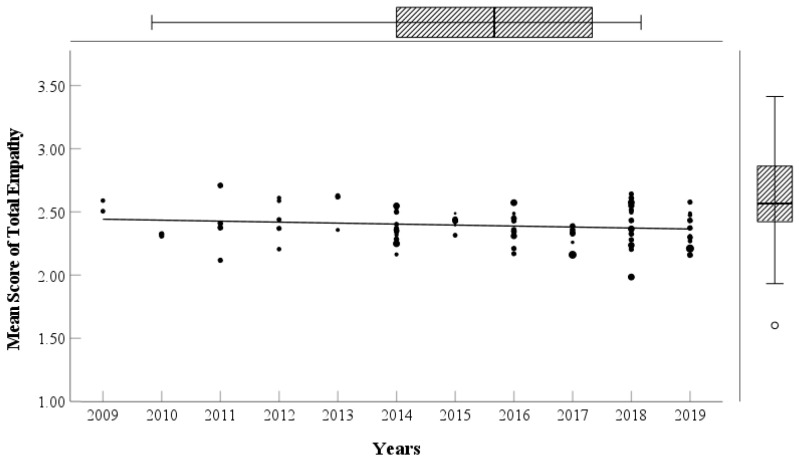
Changes in scores of Chinese college students’ total empathy from 2009 to 2019. Note: The points represent dependent variable scores from studies, and their size is proportional to each study’s log_10_(sample size). The line represents the linear fit to the data. Applies to all figures.

**Figure 3:**
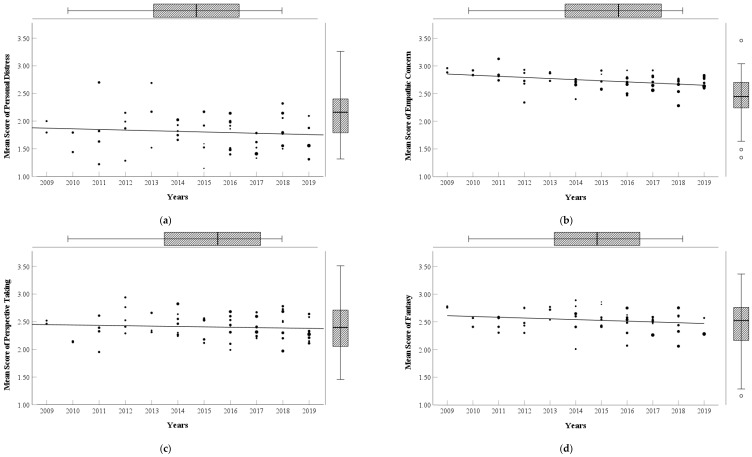
Changes in scores of empathy dimensions among Chinese college students from 2009 to 2019. (**a**) Personal distress; (**b**) empathic concern; (**c**) perspective taking; (**d**) fantasy.

This study generated scatter plots of total empathy and its dimensions with years among Chinese college students from 2009 to 2019 (see Figure 2 and Figure 3); the scores of total empathy and its dimensions gradually decrease with the years. To examine the significance of the decrease in empathy, this study conducted a sample-size-weighted regression analysis following previous research (Twenge & Campbell, 2001). The sample size weighting helped account for differences in sample sizes across the studies, minimizing their potential influence on the results. The results (see Table 3) show that sampling years significantly negatively predict personal distress (*p* = 0.029), empathic concern (*p* < 0.001), fantasy (*p* = 0.003), and total empathy (*p* = 0.008) but marginally significantly negatively predict perspective taking (*p* = 0.092).

In studies related to cross-temporal meta-analyses, researchers have particularly focused on excluding the effects of extraneous factors (Xin & Xin, 2017). For example, the regional disparities in marketization process may lead to variations in results depending on the regions from which data are collected. Additionally, differences in publication classifications could also impact the results, as journals may have varying requirements regarding samples’ quality and size. Therefore, to examine the stability of the time effect, this study conducted a sample-size-weighted regression analysis again, controlling for the effects of data collection regions and publication classes as covariates. Specifically, stepwise regression analyses were conducted separately for empathy and its dimensions as dependent variables. In the first step, publication classes and data collection regions (with the categorical variables converted to dummy variables) were included in the regression model as control variables. In the second step, sampling years were included as an independent variable in the regression model established in the previous step.

The results (see Table 3) revealed significant effects based on publication classes and data collection regions. For the publication classes, significant effects were observed in the Chinese core journals (β_PD_ = −0.46, *p*_PD_ = 0.002; β_PT_ = −0.38, *p*_PT_ = 0.008; β_FS_ = −0.44, *p*_FS_ < 0.001; β_Empathy_ = −0.57, *p*_Empathy_ < 0.001) and the English journals (β_PD_ = 0.28, *p*_PD_ = 0.028; β_FS_ = 0.37, *p*_FS_ = 0.001; β_Empathy_ = 0.29, *p*_Empathy_ = 0.004). For the data collection regions, significant effects were observed in the Western region (β_PT_ = −0.29, *p*_PT_ = 0.020), the Northeastern region (β_FS_ = −0.27, *p*_FS_ = 0.031), and the Middle region (β_Empathy_ = 0.22, *p*_Empathy_ = 0.032). However, the regression coefficients for the remaining dummy variables were not significant (*p*s > 0.05). After controlling for these factors, the sampling years remained significant predictors of personal distress (*p* = 0.005), empathic concern (*p* < 0.001), perspective taking (*p* = 0.004), fantasy (*p* < 0.001), and total empathy (*p* < 0.001). The range of ∆*R*^2^ values (0.11–0.26) collectively suggests that sampling years have medium-to-large (0.13–0.26) effect sizes in explaining the variance in empathy and its dimensions.

### 3.2. Evaluating the Magnitude of Empathy Change over Time

We calculated the magnitude of changes in total empathy and its dimensions from the first year (2009) to the last year (2019) in the data set (see Table 4). Specifically, we constructed an equation (*M* = *B*x + *C*) using the unstandardized variance coefficient (*B*) and constant (*C*). Both *B* and *C* come from the regression weighted by sample size when controlling for publication class and data collection region. Then, we respectively substituted 2009 and 2019 for x in the equation to obtain the scores (M2009 and M2019) of empathy and its dimensions in 2009 and 2019. Then the magnitudes of changes in empathy and its dimensions over time were calculated according to the equation *d* = (M2019 − M2009)/*M*_SD_; *M*_SD_ represents the averaged *SD*.

The effect sizes of the decreasing changes in total empathy and its dimensions (i.e., personal distress, empathic concern, perspective taking, and fantasy), respectively, are 0.79, 0.69, 0.49, 0.50, and 0.76 standard deviations from 2009 to 2019. Therefore, according to the guidelines (Cohen, 1992), these *d*s of total empathy and its dimensions should be considered medium-to-large (0.50–0.80) effect sizes. Overall, empathy shows a significant decline during 2009–2019.

### 3.3. Associations Between Marketization and Empathy

Marketization encompasses various aspects of economic development and varies across periods and regions. To comprehensively assess the relationship between marketization and empathy and better support the research hypothesis, this study examined their relationship from multiple perspectives, as outlined below:

#### 3.3.1. The Relationship Between Different Components of Marketization and Empathy

Marketization manifests in various aspects of economic development. This study examined the associations between different components of marketization and empathy to enhance the robustness of the findings. Regression analyses were conducted to explore the relationships between the marketization index and its components and empathy. These analyses were weighted by sample size, with total empathy and its dimensions as dependent variables, the publication category and the data collection region as control variables, and the five components of the marketization index included separately as independent variables.

The results (see Table 5) indicate that the effects of publication classes and data collection regions are significant (see Supplementary Materials Table S1). MI1 (i.e., the relationship between the government and the market) significantly and positively predicts empathic concern (*p* = 0.005), fantasy (*p* = 0.002), and total empathy (*p* = 0.002). MI2 (i.e., the development of the non-public economy) significantly and negatively predicts empathic concern (*p* = 0.008) and marginally and negatively predicts fantasy (*p* = 0.082) and total empathy (*p* = 0.065). MI3 (i.e., the development of the product market) significantly and positively predicts personal distress (*p* = 0.009), perspective-taking (*p* = 0.003), fantasy (*p* < 0.001), and total empathy (*p* = 0.001). In contrast, MI4 (i.e., the development of the factor market) and MI5 (i.e., the development of market intermediaries and the legal institutional environment) exhibit significant negative associations with empathy and all its dimensions (*p*s < 0.01). These findings suggest that different components of marketization are associated with empathy and its dimensions to varying degrees. In summary, MI1 and MI3 exhibit positive correlations with empathy, whereas MI2, MI4, and MI5 show negative correlations. To integrate the effects of different aspects of marketization on empathy, the five components of the marketization index were aggregated into an overall marketization index and used to predict empathy and its dimensions. The results indicate a stable and consistent negative predictive effect of total marketization index on empathy and its dimensions (*p*s < 0.05).

#### 3.3.2. The Relationship Between Marketization and Empathy from a Temporal Perspective

The method of cross-temporal meta-analysis can reveal the relationship between social change and individual development. The emergence of individual psychological developmental characteristics may occur later than the emergence of the social events that cause them to change. A lag regression analysis method can reveal a temporal association between the level of marketization and empathy, relying on the temporal order of the independent and dependent variables. Thus, the present study conducted lag regression analyses to explore the correlation between the marketization index of 5 years prior, 3 years prior, 1 year prior, and the current year with total empathy and its dimensions. These regression analyses were weighted by the sample sizes, based on total empathy and its dimensions as the dependent variables, the publication classes and data collection regions as the control variables, and the marketization index as the independent variable.

The results (see Table 5) illustrate that the effects of publication classes and data collection regions are significant (see Supplementary Materials Table S1). The marketization index for the year of the empathy data collection, as well as one year and five years prior, significantly negatively predicted total empathy and its dimensions (*p*s < 0.05). Additionally, the marketization index from three years prior also significantly negatively predicted empathic concern (*p* < 0.001), fantasy (*p* < 0.001), and total empathy (*p* = 0.004). This indicates that the marketization index at earlier time points significantly negatively predicts empathy in the following years. Overall, these findings provide evidence of a temporal association between marketization and empathy, further supporting the research hypothesis that empathy may weaken over time within the context of marketization.

#### 3.3.3. The Relationship Between Marketization and Empathy from a Spatial Perspective

In China, the degree of marketization varies across regions, providing an opportunity to analyze the relationship between marketization and empathy based on these regional differences. If the negative predictive effect of the marketization index on empathy is weaker in regions with lower marketization (compared to regions with higher marketization), it would provide stronger support for the notion that empathy may weaken with the development of marketization. Thus, the present study conducted regression analyses, including the interaction term between the marketization index and the data collection regions, to explore regional differences in the predictive effects of the marketization index on empathy. These regression analyses were weighted by the sample sizes and used total empathy and its dimensions as the dependent variables, with the publication class as the control variable, and the interaction term between the marketization index and the data collection regions as the independent variable.

The results (see Table 5) indicate that the effects of publication classes and data collection regions are significant (see Supplementary Materials Table S1). The marketization index (MI) significantly negatively predicts total empathy and its dimensions (*p*s < 0.05) in the reference group (i.e., the Eastern region). This suggests that the negative association between marketization and empathy holds in the Eastern region. The further analysis of regional differences reveals that, compared to the Eastern region, the negative predictive effect of the MI on empathy and its dimensions is generally weaker in other regions, with some reaching statistical significance.

For example, in the Northeastern region, the negative predictive effect of the MI on fantasy (β_MI in Northeastern_ = β_MI_ + β_MI × DCR [Northeastern]_ = −3.11 + 2.97 = −0.14) is significantly weaker than that in the Eastern region, with a significant difference (β_MI × DCR [Northeastern]_ = 2.97, *p* = 0.043) from the strong negative effect (β_MI_ = −3.11) observed in the Eastern region. Similarly, the negative predictive effect of the MI on total empathy in the Northeastern region (β_MI in Northeastern_ = −0.91) is marginally weaker (*p* = 0.092) than in the Eastern region. A similar pattern was observed in the Western region, where the negative predictive effect of the MI on fantasy (β_MI in Western_ = −0.65) is significantly weaker than in the Eastern region (*p* = 0.033), and its negative predictive effect on total empathy (β_MI in Western_ = −1.12) is marginally weaker (*p* = 0.071) than in the Eastern region. For the unreported or cross-regional group, where the MI was assigned the national average marketization index, the MI positively predicts personal distress (β_MI in unreported_ = 0.68). This effect is marginally significant (*p* = 0.083) and differs from the significant negative predictive effect of the MI on personal distress observed in the Eastern region (β_MI_ = −1.77). Additionally, the predictive effects of the MI on perspective taking (β_MI in unreported_ = 0.65), fantasy (β_MI in unreported_ = −0.53), and total empathy (β_MI in unreported_ = 0.03) also show significant differences from those in the Eastern region (*p*s < 0.05).

Overall, this study found a stronger negative predictive effect of the marketization index on empathy and its dimensions in highly marketized regions (i.e., the Eastern region). This finding further supports the notion that empathy may weaken with the development of marketization.

## 4. Discussion

### 4.1. Changing Trends in Empathy Among Chinese College Students

The present cross-temporal meta-analysis shows the downward trend in empathy among Chinese college students between 2009 and 2019. This cross-temporal meta-analysis has more abundant research articles and a broader period, thus the result of the observed empathy change is more robust, better observable, and more credible. In addition, some studies further support this result. Empathy and its dimension of perspective taking decreased among Chinese nurses from 2009 to 2018 (Xu et al., 2021); a study of empathy changes among American college students showed that empathic concern and perspective taking dropped significantly between 1979 and 2009 (Konrath et al., 2011). A similar trend of empathy decreasing over time has been found across different groups and cultures, revealing that we should discuss empathy change from a more macro perspective.

Decreased empathy may be a sign of social moral change at the cohort level. Among the much-discussed theories of moral change in Chinese society, the moral decline model is prominent, which suggests that moral declines over time during social change (Sun, 2007). For example, since 1990, Chinese people have increasingly accepted the competition to obtain wealth and became more tolerant of unethical financial activities (Xin & Li, 2020). Given the fundamental role of empathy in morality, the result of the decline over time in empathy also somewhat supports the moral decline model.

### 4.2. The Relationship Between Marketization with Empathy and Its Theoretical Explanation

The present study found that the empathy of Chinese college students decreased as the marketization index increased, which suggests that the social change theory is appropriate to explain the decline of empathy. Zarins and Konrath (2017) had a similar view. They tried to adopt Greenfield’s (2009) theory of social change and human development to elucidate the decrease in empathy in the United States. Specifically, they argued that changes in societal structures may lead to cultural shifts, such as an increased self-focus and a decreased other-focus. However, Zarins and Konrath did not present economic-related macro factors that caused the change in empathy. The IAT is another helpful theory in understanding the effect of marketization on empathy. It focuses on the negative impact of the macro-market economic system on the micro-individual and claims that institutional imbalances in the form of economic dominance increase crime rates in society through anomic values (Messner et al., 2019). Furthermore, Groß et al. (2018) included individuals’ lower commitment to self-transcendence via altruism (e.g., being touched by the destiny of other people, sympathizing with and understanding other people) as a dimension of marketized mentality, which is an individual-level concept of the anomic culture depicted in the IAT. In conclusion, we integrate the perspective of social-ecological psychology (Uskul & Oishi, 2020) and suggest that the decline in empathy at the group level may temporally co-vary with macro-level marketization development.

Since empathy is an antecedent of social morality, the negative association between marketization and empathy implies that it is necessary to be alert to the destructive impact of marketization on social morality. Of particular relevance for this is the study in which Xin (2019) examined marketization’s social consequences based on the dualism of human nature. Concretely, the market economy activated “economic man” beliefs but weakened “social man” characteristics: people tended to pursue their own interests and reduced their trust in others. The assumption of economic man beliefs in economics originates from Smith’s view in *The Wealth of Nations*. Smith (1776/2023) claimed that self-interest is the fundamental motivation for people to participate in social and economic activities. In Smith’s (1790/2010) repeatedly revised work, *The Theory of Moral Sentiments*, he emphasized that sympathy is the primordial altruistic instinct of all people and is the basis of virtue. This virtue can effectively restrain people’s excessive pursuit of personal wealth and fame. To sum up, Smith’s view of human nature is dualistic, in which self-interest and other interests coexist.

This study tried to use Smith’s reported concept of the dualism of human nature to analyze social morality’s erosion by marketization. We found that marketization and self-interest (economic man) beliefs can promote each other in a vicious spiral. The profit-seeking values created in marketization and individuals’ frequent economic activities can both strengthen their economic man beliefs (Messner et al., 2019). In turn, individuals’ increasingly inflated self-interest tendencies can drive them to further engage in market activities. Moreover, the present study found that marketization reduces empathy. The market institution also reduces individuals’ social preferences (Reeson & Tisdell, 2010). These findings imply that marketization can also weaken the internal restriction mechanism (the altruistic nature of empathy) of self-interest. Once individual self-interest beliefs break through the market space and enter the social sphere, they may impact civic morality to some degree.

### 4.3. Implications and Limitations

The theoretical value of this study is based on Smith’s concept of the dualism of human nature, which we incorporated to expand Xin’s (2019) view of marketization inhibiting interpersonal trust. Concretely, we further argue for the recognition of the negative relationship between marketization and empathy, and aim to enrich the relevant research on the social consequences of marketization. The practical implication of this study is to warn that while people enjoy the developmental fruits of market-oriented reforms, they must be alert to the erosion of marketization on social morality (Klein, 2016; Teo, 2018). Besides, the present study provides inspiration for the practice of social governance to restrict the adverse effects of marketization on social morality by increasing individuals’ levels of empathy. For instance, family parenting develops children’s ability to understand and pity others; school education improves students’ critical thinking skills and their ability to put themselves in others’ positions.

However, this study also has several limitations that must be acknowledged: First, this study only focused on the relationship between marketization and empathy. Many other sociocultural and economic factors, such as technological advancements, urbanization, and educational reforms, may also influence empathy levels. Future research could incorporate a broader range of predictive variables to provide a more comprehensive understanding of empathy trends. Second, this study was conducted solely with Chinese college students, which limits the generalizability of the findings to the broader population. College students may differ from other groups in terms of their socioeconomic status, life experience, and exposure to market forces. Therefore, caution is needed when generalizing these results to the general public. Future studies should include more diverse samples from different age groups, educational backgrounds, and regions. Third, this study only included research that measured empathy using the IRI-C scale, potentially overlooking studies that employed other valid empathy measurement tools. Future research should include various measurement tools to provide stronger evidence of empathy trends.

## 5. Conclusions

This study found that Chinese college students’ empathy has declined over time within the context of marketization.

## Figures and Tables

**Table 1:** Distribution of study data in cross-temporal meta-analysis, 2009–2019.

Sampling Years	Number of Samples	Sample Size
2009	2	410
2010	2	728
2011	4	1904
2012	5	1047
2013	3	743
2014	12	5921
2015	8	2697
2016	11	5346
2017	10	8661
2018	15	9226
2019	17	11,717

**Table 2:** Descriptive statistics of empathy.

Dimensions	Number of Samples	Sample Size	*M* ± *SD*
Personal distress	55	30,972	1.80 ± 0.34
Empathic concern	63	36,739	2.73 ± 0.15
Perspective taking	64	37,368	2.40 ± 0.22
Fantasy	54	30,528	2.53 ± 0.20
Total empathy	79	41,560	2.39 ± 0.14

Note: The number of samples varies from 54 to 79, and the sample size varies from 30,528 to 41,560. The reason is that some of the studies only reported the data of some empathy dimensions or total empathy.

**Table 3:** The relationship of sampling years with empathy.

Dimensions	Sample Size Weighted				Sample Size Weighted with Covariates			
	β	*t*	*p*	*R* ^2^	β	*t*	*p*	∆*R*^2^
Personal distress	−0.29(55)	−2.24	0.029	0.09	−0.41(55)	−2.92	0.005	0.11
Empathic concern	−0.44(63)	−3.80	<0.001	0.19	−0.50(63)	−3.81	<0.001	0.18
Perspective taking	−0.21(64)	−1.71	0.092	0.05	−0.39(64)	−3.04	0.004	0.11
Fantasy	−0.39(54)	−3.09	0.003	0.16	−0.64(54)	−5.17	<0.001	0.26
Total empathy	−0.30(79)	−2.74	0.008	0.09	−0.51(79)	−5.18	<0.001	0.20

Note. The number of samples is shown in parentheses.

**Table 4:** Evaluating the magnitude of changes in empathy, 2009–2019.

Dimensions	Equation	*M*_2019_	*M*_2009_	*M*_SD_	*d*
Personal distress	*y* = 103.817 − 0.051*x*	0.85	1.36	0.74	−0.69
Empathic concern	*y* = 58.695 − 0.028*x*	2.16	2.44	0.57	−0.49
Perspective taking	*y* = 69.163 − 0.033*x*	2.54	2.87	0.66	−0.50
Fantasy	*y* = 101.355 − 0.049*x*	2.42	2.91	0.65	−0.76
Total empathy	*y* = 69.326 − 0.033*x*	2.70	3.03	0.42	−0.79

**Table 5:** The relationship of the marketization index with empathy.

Variables	Personal Distress				Empathic Concern				Perspective Taking				Fantasy				Total Empathy			
	β	*t*	*p*	∆*R*^2^	β	*t*	*p*	∆*R*^2^	β	*t*	*p*	∆*R*^2^	β	*t*	*p*	∆*R*^2^	β	*t*	*p*	∆*R*^2^
(1) Different components of marketization and overall marketization																				
MI1	0.32	1.48	0.146	0.03	0.74	2.94	0.005	0.12	0.33	1.27	0.210	0.02	0.66	3.38	0.002	0.14	0.56	3.03	0.003	0.08
MI2	−0.42	−1.02	0.311	0.02	−0.96	−2.74	0.008	0.10	−0.45	−1.27	0.211	0.02	−0.77	−1.78	0.082	0.05	−0.54	−1.87	0.065	0.03
MI3	0.41	2.73	0.009	0.10	0.12	0.71	0.481	0.01	0.44	3.13	0.003	0.12	0.59	4.05	<0.001	0.19	0.43	3.48	<0.001	0.10
MI4	−0.91	−2.86	0.006	0.11	−0.90	−3.18	0.002	0.13	−0.89	−3.33	0.002	0.13	−1.41	−4.87	<0.001	0.24	−1.02	−5.10	<0.001	0.19
MI5	−0.77	−2.82	0.007	0.10	−0.58	−2.75	0.008	0.10	−0.59	−3.04	0.004	0.11	−1.37	−5.25	<0.001	0.27	−0.83	−4.58	<0.001	0.16
MI	−0.97	−2.19	0.033	0.07	−1.55	−3.72	<0.001	0.17	−1.10	−2.62	0.011	0.09	−1.46	−3.46	0.001	0.15	−1.27	−4.16	<0.001	0.14
(2) A temporal perspective																				
MI_Current year	−0.97	−2.19	0.033	0.07	−1.55	−3.72	<0.001	0.17	−1.10	−2.62	0.011	0.09	−1.46	−3.46	0.001	0.15	−1.27	−4.16	<0.001	0.14
MI_One year prior	−0.98	−2.15	0.037	0.06	−1.50	−3.74	<0.001	0.18	−1.02	−2.53	0.014	0.08	−1.83	−4.28	<0.001	0.20	−1.31	−4.10	<0.001	0.14
MI_Three years prior	−0.76	−1.55	0.129	0.03	−2.02	−4.17	<0.001	0.21	−0.71	−1.38	0.172	0.03	−1.67	−3.77	<0.001	0.17	−1.00	−3.02	0.004	0.08
MI_Five years prior	−0.96	−2.11	0.041	0.06	−1.37	−2.95	0.005	0.12	−1.31	−2.97	0.004	0.11	−1.87	−4.76	<0.001	0.24	−1.35	−4.23	<0.001	0.14
(3) A spatial perspective																				
MI	−1.77	−2.06	0.046	0.14	−2.28	−2.52	0.015	0.25	−2.50	−2.72	0.009	0.16	−3.11	−3.97	<0.001	0.24	−2.72	−4.05	<0.001	0.22
MI × DCR [Middle]	0.00	0.00	0.999		−0.58	−0.49	0.627		1.35	1.12	0.268		1.38	1.35	0.185		1.00	1.18	0.243	
MI × DCR [Northeastern]	2.34	1.50	0.141		0.67	0.41	0.685		1.66	0.99	0.326		2.97	2.09	0.043		1.81	1.71	0.092	
MI × DCR [Western]	0.77	0.66	0.514		1.77	1.56	0.126		1.28	1.10	0.278		2.46	2.21	0.033		1.60	1.83	0.071	
MI × DCR [unreported...]	2.45	1.78	0.083		1.42	1.05	0.301		3.15	2.40	0.020		2.58	2.06	0.046		2.75	3.17	0.002	

Note. (1) MI refers to the marketization index, while MI1 represents one of its components, namely the relationship between the government and the market. MI2 corresponds to the development of the non-public economy, MI3 to the development of the product market, MI4 to the development of the factor market, and MI5 to the development of market intermediaries and the legal institutional environment. (2) MI_Current year refers to the marketization index for the year in which the empathy data were collected. MI_One year prior represents the marketization index from one year before data collection, MI_Three years prior corresponds to the index from three years before data collection, and MI_Five years prior indicates the marketization index from five years before data collection. (3) DCR is the abbreviation for the categorical variable “data collection regions”. Given that the marketization index in the Eastern region is significantly higher than that of other regions, as well as the national average, which corresponds to the unreported or cross-regional group (*p*s < 0.001), the regression analysis incorporating the interaction term MI × DCR designated the Eastern region as the reference group.

## Data Availability

The references and data of the cross-temporal meta-analysis were shared in the public repository OSF https://osf.io/wrhnu/?view_only=8e025eed7b044d038633cf171fdfdbc1 (accessed on 2 January 2025).

## Note

1 The IRI-C was translated and revised by Chan (1986) based on the IRI developed by Davis (1980). The factor analysis of the Chinese version revealed a four-factor model consistent with Davis’s original study, with one to two items removed from each factor due to cultural differences. The final IRI-C, like the original IRI, includes four dimensions: personal distress, which reflects experiencing one’s own negative emotions when witnessing another person’s distress (five items); empathic concern, which reflects the desire to help or comfort others in need (six items); perspective taking, which reflects taking another person’s perspective and understanding their thoughts and feelings (five items); and fantasy, which reflects perceiving the emotions of fictional characters (six items). This scale is widely used in Chinese empathy research, having been cited 673 times in CNKI as of January 2025 and demonstrating good reliability and validity (Zhang et al., 2010).

## Supplementary Materials

The following are available online at: https://www.mdpi.com/article/10.3390/bs15050597/s1.
